# The risks of pityriasis rosea in pregnancy: a review

**DOI:** 10.1097/JW9.0000000000000191

**Published:** 2025-01-16

**Authors:** Sophia Manduca, Christina S. Oh, Michael Ong, Shari R. Lipner, Miriam K. Pomeranz, Amy K. Bieber

**Affiliations:** a The Ronald O. Perelman Department of Dermatology, New York University Grossman School of Medicine, New York City, New York; b Department of Dermatology, Weill Cornell Medicine, New York City, New York; c NYC Health + Hospitals/Bellevue Hospital Center, New York City, New York

**Keywords:** complications, human herpesvirus, pityriasis rosea, pregnancy

## Abstract

**Objective:**

This review aims to consolidate available evidence, identify research gaps, and advocate for a more informed approach to the management of pityriasis rosea in pregnant individuals.

**Data Sources:**

PubMed, Web of Science, and Directory of Open Access Journals were systematically searched based on the keywords “pityriasis rosea,” “pityriasis circinate,” “roseola annulate,” “herpes tonsurans maculosus,” “herald patch,” and “pregnancy” on January 25, 2024 for publications between 1950 to 2024.

**Study Selection:**

Studies containing outcomes data for pregnant patients with established PR were included. Studies must have been written or translated into English and published in a peer-reviewed journal. Studies which did not pertain to PR in the setting of pregnancy were excluded, as screened by two reviewers. Responses, general informational reviews, and letters to the editor without novel data were also excluded.

**Results:**

Eleven relevant articles were identified, encompassing data from 177 patients. Overall, 81% of patients had favorable outcomes while 19% experienced unfavorable outcomes. PR onset before 15 weeks gestation was associated with a higher rate of unfavorable outcomes (41%), including a 27% rate of spontaneous abortion (SA). Conversely, PR onset after 15 weeks had a lower unfavorable outcome rate (21%), and no instances of SA.

**Conclusion:**

Conflicting data exists regarding the impact of PR on pregnancy outcomes. However, PR onset within the first 15 weeks, widespread lesions, constitutional symptoms, and higher human herpesvirus 6 viral loads may increase the risk of unfavorable outcomes such as SA. Close follow-up and consideration of antiviral treatment are recommended for high-risk patients.

**What is known about this subject in regard to women and their families?**

- Pityriasis rosea (PR) is a self-limited skin condition often associated with the reactivation of human herpesvirus 6 and 7.
- Historically, PR is more prevalent in pregnant women, likely due to a state of immunosuppression.
- There is conflicting evidence in the current literature: some studies have shown that PR in pregnancy, particularly when it occurs within the first 15 weeks, may lead to unfavorable birth outcomes like spontaneous abortion (SA), whereas other studies have countered this claim.

**What is new from this article as message for women and their families?**

- This article differs from existing research in that we conduct a thorough review of the literature and reanalyze previously reported outcomes based on the timing of rash onset during gestation to make relevant clinical recommendations. We present a comparative analysis of PR onset before versus after 15 weeks of pregnancy, the former of which has been suggested in the literature to herald less desirable pregnancy outcomes.
- We present the notion that, outside of SA, initially observed unfavorable pregnancy outcomes associated with PR infection may be temporary. Given the transient nature of most of the adverse effects, we advocate that the main concern for this patient population would be increased rates of SA.

## Introduction

Pityriasis rosea (PR) is a self-limited papulosquamous eruption characterized by pink, ovoid or round, small patches or plaques with fine scale on the trunk and proximal extremities. Classically, these lesions are oriented along relaxed skin tension lines, giving a characteristic “Christmas tree” pattern. The eruption is often preceded by one larger lesion termed the “herald patch.” The rash may be asymptomatic or pruritic and usually lasts 2 to 8 weeks. While often absent, systemic symptoms may include nausea, anorexia, malaise, headache, fever, arthralgia, and lymphadenopathy.^1,2^ The exact etiology of PR is unknown, though the leading hypothesis is that PR is related to reactivation of human herpesvirus (HHV) 6 and 7 with numerous studies documenting this association.^3–6^

The differential diagnosis of PR includes secondary syphilis, seborrheic dermatitis, erythema multiforme, tinea corporis, and a PR-like medication reaction.^7^ Syphilis, an important differential diagnosis that poses serious risk to the fetus, must be ruled out in all pregnant patients presenting with PR.^8^ It is recommended that the specific dermatoses of pregnancy be considered any time a pregnant woman presents with a new rash; these include atopic eruption of pregnancy and polymorphic eruption of pregnancy, which pose no risk to the fetus, as well as pemphigoid gestationis and intrahepatic cholestasis of pregnancy, which do pose risk to the fetus.^6,7^ PR may present atypically in up to 20% of patients, including as vesicular, follicular, inverse, and acral variants of PR.^8^

In the general population, the approximate incidence of PR is 0.5 to 2%.^8^ PR has historically been reported as more prevalent in pregnant individuals,^9^ which is thought to be due to a state of immunosuppression that increases the risk of viral illnesses.^6,9,10^ In 2008, one study showed an association between adverse birth outcomes and PR occurring during pregnancy, including an alarming rate (62%) of spontaneous abortion (SA) in women who developed PR within the first 15 weeks of pregnancy.^11^ Numerous studies have since been conducted to further elucidate the risks of PR in pregnancy. Herein we review those studies.

## Methods

**Fig. 1.**
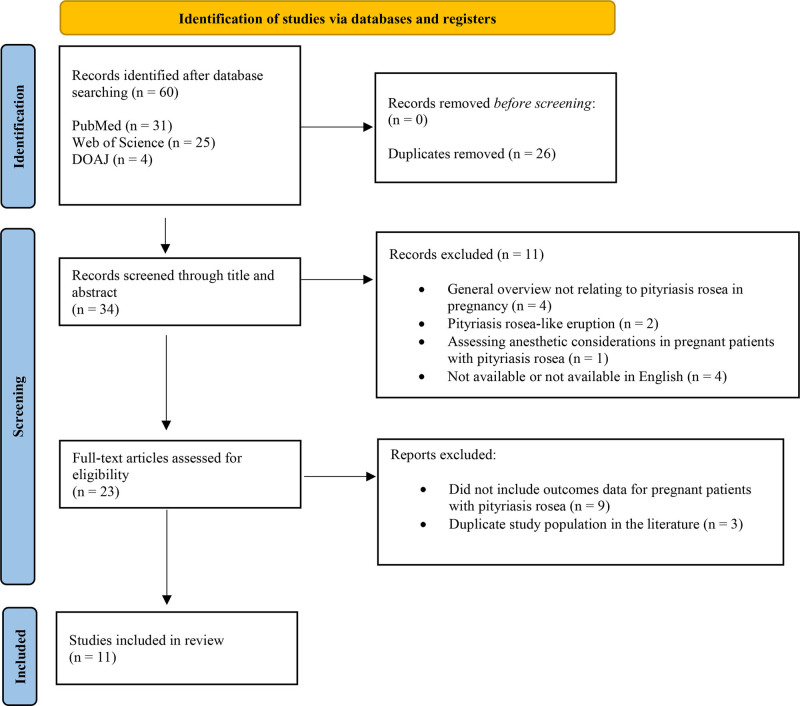
Study selection.

PubMed, Web of Science, and Directory of Open Access Journals were systematically searched based on the keywords “pityriasis rosea,” “pityriasis circinate,” “roseola annulate,” “herpes tonsurans maculosus,” “herald patch,” and “pregnancy” on January 25, 2024, for publications between 1950 and 2024. After the removal of duplicate articles, articles were screened for eligibility based on pre-established study criteria. Studies that reported outcomes data for pregnant patients with established PR were included. Studies must have been written in or translated into English and published in a peer-reviewed journal. Studies that did not pertain to PR in the setting of pregnancy were excluded, as screened by 2 reviewers (Fig. 1). Any discrepancies were resolved by discussion between reviewers. Responses, general informational reviews, and letters to the editor without novel data were also excluded. For all selected articles, references were also reviewed to identify further relevant studies. Individual patient data were extracted from each selected article and compiled in Tables 1 and 2. In this analysis, criteria for an unfavorable pregnancy outcome were: SA, preterm delivery before 37 weeks of gestation, birth weight less than 2,500 grams, and meconium passage before delivery.

**Table 1:** Characteristics and outcomes of all 177 patients with PR in pregnancy

Study source	Total number of patients	Mean age	Favorable outcome	Unfavorable outcome (excluding SA)	SA	Key findings
Drago et al. 2008^11^	38	29.3	24 (63%)	9 (24%)	5 (13%)	In patients who developed PR within the first 15 weeks of pregnancy, a significantly increased spontaneous abortion rate was noted. PR may also be associated with HHV 6 infection.
Drago et al. 2014^12^	23^a^	30.6	17 (74%)	3 (13%)	3 (13%)	Replicated previous findings as above.
Drago et al. 2018^13,^^b^	NA	NA	NA	NA	NA	Physicians should take clinical signs and HHV 6 viral load into consideration in pregnant women with pityriasis rosea.
Chuh et al. 2005^14^	2	30.5	2 (100%)	0	0	Uneventful pregnancies despite PR onset.
Bianca et al. 2007^2^	1	32	1 (100%)	0	0	Uneventful pregnancy despite PR onset. Syphilis is an important differential diagnosis to be ruled out when PR is suspected.
Loh et al. 2016^15^	1	28	1 (100%)	0	0	Occurrence of isolated craniosynostosis in a patient with early onset PR.
Cruz et al. 2011^16^	1	28	1 (100%)	0	0	Uneventful pregnancy despite PR onset. PR associated with HSV 2 in one patient.
Karaer et al. 2012^17^	1	28	0	1 (100%)	0	Preterm birth in a patient with first trimester PR onset with history of a prior preterm birth.
Stashower et al 2021^18^	33^c^	26.8	30 (91%)	3 (9%)	0	In a cohort of pregnant women with PR, no spontaneous abortions nor increased unfavorable outcomes compared to the general population were observed.
Wenger-Oehn et al., 2022^19,^^d^	46	28	41 (89%)	3 (7%)	2 (4%)	Early onset, extensive spread, long rash duration, and presence of extracutaneous symptoms were directly associated with an unfavorable pregnancy outcome in women with PR.
Ong et al. 2024^20,^^e^	31	32.9	26 (84%)	1 (3%)	4 (13%)	Pregnant patients with PR did not exhibit a higher risk of complications compared to matched controls.
Total	177	29.5	143 (81%)	20 (11%)	14 (8%)	

Compiled data of 177 patients from all appraised studies. Criteria for an unfavorable pregnancy outcome were: spontaneous abortion (SA), preterm delivery before 37 weeks of gestation, birth weight less than 2,500 grams, and meconium passage before delivery. HHV, human herpesvirus; PR, pityriasis rosea; SA, spontaneous abortion.

a This study included 23 new patients, all other patients were previously included in Drago et al. 2008 analysis. Individual data was only presented for 9 new patients with unfavorable outcomes and/or perinatal problems as defined by the authors, though favorable outcomes data were extrapolated.

b Individual data not presented for new patients, all other patients previously included in prior Drago et al. publications.

c Individual data only presented for 8 patients with birth complications as defined by the authors. Favorable outcomes data were extrapolated to include in Table 1.

d Individual data regarding PR onset not available. As such, this data was not included in calculations related to temporal onset of PR (Table 2), though was included in overall outcomes data (Table 1).

e Individual data regarding PR onset was generously provided by the research team.

**Table 2:** Temporal assessment of PR onset and pregnancy outcomes

Study source	Total number of patients	Mean age	Outcomes: PR within first 15 weeks of pregnancy			Outcomes: PR after 15 weeks of pregnancy		
			Favorable	Unfavorable excluding SA	SA	Favorable	Unfavorable excluding SA	SA
Drago et al. 2008^11^	38	29.3	3 (38%)	0	5 (62%)	21 (70%)	9 (30%)	0
Drago et al. 2014^12^	9^a^	30.6	1 (14%)	3 (43%)	3 (43%)	2 (100%)	0	0
Drago et al. 2018^13,^^b^	NA	NA	NA	NA	NA	NA	NA	NA
Chuh et al. 2005^14^	2	30.5	1 (100%)	0	0	1 (100%)	0	0
Bianca et al. 2007^2^	1	32	1 (100%)	0	0	0	0	0
Loh et al. 2016^15^	1	28	1 (100%)	0	0	0	0	0
Cruz et al. 2011^16^	1	28	0	0	0	1 (100%)	0	0
Karaer et al. 2012^17^	1	28	0	1 (100%)	0	0	0	0
Stashower et al 2021^18^	8^c^	26.75	3 (60%)	2 (40%)	0	3 (100%)	0	0
Wenger-Oehn et al. 2022^19,^^d^	NA	NA	NA	NA	NA	NA	NA	NA
Ong et al. 2024^20,^^e^	31	32.9	16 (80%)	0	4 (20%)	10 (91%)	1 (9%)	0
Total	92	29.5	26 (59%)	6 (14%)	12 (27%)	38 (79%)	10 (21%)	0 (0%)

Compiled data of 92 patients for whom temporal data regarding PR onset was available (PR onset within versus after the first 15 weeks of pregnancy). Criteria for an unfavorable pregnancy outcome were: spontaneous abortion (SA), preterm delivery before 37 weeks of gestation, birth weight less than 2,500 grams, and meconium passage before delivery. PR, pityriasis rosea; SA, spontaneous abortion.

a Only 9 new patients in this expanded series, all other patients previously included in Drago et al. 2008 analysis. Individual data, including PR onset, was only presented for patients with unfavorable outcomes and/or perinatal problems as defined by the authors.

b Individual data not presented for new patients, all other patients previously included in prior Drago et al. publications.

c Individual data only presented for 8 patients with birth complications as defined by the authors.

d Individual data regarding PR onset not available. As such, this data was not included in calculations related to temporal onset of PR (Table 2), though was included in overall outcomes data (Table 1).

e Individual data regarding PR onset was generously provided by the research team.

## Results

An initial search of PubMed, Web of Science, and Directory of Open Access Journals generated 60 peer-reviewed articles. After 26 duplicates were removed, abstracts and titles of all studies identified by the search strategy were reviewed. Of the 34 articles, 11 articles were excluded as detailed in Figure 1, leaving an initial total of 23 articles. The reference lists of the 23 included articles were reviewed, which yielded no additional articles that fit the inclusion criteria. Papers that did not include outcome data for pregnant patients with established PR were excluded. A total of 11 relevant articles met the selection criteria for this review.

A total of 177 patients were analyzed. In all 177 patients with PR during pregnancy, overall favorable outcomes occurred in 143 patients (81%), and unfavorable outcomes in 34 patients (19%), of which 14 (8%) were SA. Data on PR onset was available for 92 patients, and only those patients were included for subanalysis by week of onset. In patients with PR onset before 15 weeks gestation, 18 (41%) had unfavorable outcomes, of which 12 (27%) represented SA. In patients with PR onset after 15 weeks gestation, 10 (21%) had unfavorable outcomes, of which 0% represented SA. Results are summarized in Table 2.

## Discussion

Historically, PR has not been considered more concerning in pregnant women than in the general population. In 2008, however, an observational study by Drago et al.^11^ highlighted an association between unfavorable birth outcomes—premature delivery and SA—in patients who developed PR during pregnancy. Of the 38 women in their series who developed PR during pregnancy, 9 (24%) delivered prematurely and 5 experienced SA (13%). Specifically analyzing women who developed PR within the first 15 weeks of pregnancy, 5 out of 8 patients (62%) had an SA before 16 weeks gestation, which is significantly higher than the population incidence of 10 to 15% (80% of which occur before 12 weeks gestation).^21^ These patients showed no other risk factors for SA. All women who had a SA were found to have measurable IgG antibodies against both HHV 6 and HHV 7, but not IgM, suggesting possible reactivation of these viruses.

Drago et al.^12^ later extended the aforementioned study with an additional 23 patients clinically diagnosed with PR to increase the statistical power. Of 61 patients, 8 patients spontaneously aborted (13.1%), of which 3 were in the first trimester, and 14 experienced other unfavorable outcomes (23%) characterized as hypotonia, weak motion, patent foramen ovale, hydramnios, or low birth weight. Similar to the previous study, in patients who developed PR during the first 15 weeks of pregnancy, 57% suffered a confirmed SA before 18 weeks, again significantly higher than the population incidence.^21^ All patients who spontaneously aborted were noted to have more severe PR, characterized as being unusually widespread, of long duration, and having constitutional symptoms. Outside of having PR, no risk factors for intrauterine fetal death or SA were found for these patients. All 61 patients in the study had detectable HHV 6 and 7 IgG antibodies, and none were found with detectable IgM antibodies, indicating that a recent infection was less likely. Notably, all premature neonates in this study ultimately adhered to standard growth trajectories, suggesting that complications in this cohort, outside of SA, may be more transient in nature.^12,22^

In another study, Drago. et al.^13^ sought to determine the relevant risk factors for adverse pregnancy outcomes in women with PR. Seventy-six patients with PR during their pregnancies were analyzed, of which 16 were excluded due to having known risk factors for intrauterine fetal demise. Viral loads of HHV 6 and 7 were analyzed in 50 of the patients. Of the 60 patients studied, 50% developed pregnancy complications, including SA, hydramnios, neonatal hypotonia, low birth weight and Apgar score, and patent foramen ovale. Of these complications, 8 (13%) represented SA. In patients with birth complications, the average week of PR onset was 14.8 ± 3.7, whereas in those without any complications, it was week 24.8 ± 3.6 (*P* < .001). The presence of enanthem (*P* < .04), constitutional symptoms (*P* < .001), and PR lesions over 50% of the total body surface area (*P* < .004) were also significantly associated with pregnancy complications. Of note, 5 patients with PR onset before 20 weeks were treated with oral acyclovir; 2 of those women had uncomplicated births, and 3 of these patients had complications that were unspecified by the authors. HHV 6 viral DNA loads were significantly higher in patients with complications compared to those without, at 585.15 ± 459.17 and 73.73 ± 67.82 copies/mL respectively (*P* < .001). The HHV 7 viral loads were not found to be significantly different between the 2 groups.

In a retrospective review by Wenger-Oehn et al.,^19^ 59 pregnant individuals who presented with PR at the Medical University of Graz from 2003 to 2018 were found to have a total of 63 births and SAs. Forty-six patient data sets were considered complete and included for analysis. This review also pooled their data with 53 patients from Drago et al.’s studies and 5 case studies conducted in Lebanon, China, Portugal, and the USA. Similar to Drago et al.’s findings, an earlier PR onset (*P* = .03), longer-lasting PR symptomology (*P* = .003), extended exanthem distribution (*P* = .004), and the presence of additional extracutaneous symptoms (*P* = 0.003) were significantly associated with unfavorable outcomes. In contrast, only 10.9% of the nonpooled patients resulted in an unfavorable pregnancy outcome, 2 of which were SAs. Of note, this group excluded events considered “nonsevere,” including transient weak motility, hypotonia, and hydramnios from their definition of adverse outcomes unlike Drago et al.^1,11–13^ In the nonpooled data, earlier onset, wider exanthem distribution, and constitutional symptoms trended toward being more common in patients with unfavorable outcomes, though these findings were also not statistically significant within the small sample size (*P* = .27, *P* = 063, *P* = .11, respectively). Notably, all newborns in the study population had an Apgar score of ≥8 after 5 minutes, again suggesting that sequelae outside of SA in this cohort may be temporary. Additionally, 16 of the patients had anti-HHV 6 titers measured. Of these patients, 15 (94%) had slightly to moderately elevated IgG antibodies. Thirty-four patients at the Medical University of Graz were prescribed short-term topical steroids with the remainder receiving oral antihistamines, moisturizing ointments, or no treatment.

Multiple case reports in the literature have reported no adverse pregnancy outcomes. Chuh et al.^14^ reported 2 patients with PR with normal pregnancy outcomes. Of note, only 1 patient was diagnosed with PR in the first trimester, and she had a mild presentation. Four other case reports have described women with PR during pregnancy with healthy newborns, though one had isolated craniosynostosis and another premature birth.^2,15–17^ Of those 4 reported patients, 3 had PR diagnosed in the first trimester.

Furthermore, a multicenter retrospective study by Stashower et al.^18^ evaluated medical records from 3 different centers to identify 33 pregnant patients with concurrent PR diagnosed by a dermatologist. Of these patients, only 8 (24%) were reported to have birth complications. These included 2 preterm deliveries, as well as other birth complications considered relatively minor by the authors—small for gestational age, oligohydramnios, fetal distress, chorioamnionitis, fetal oxygen resuscitation, meconium passage before delivery, nuchal cord, and retained products of conception. Notably, 7 of 33 patients were treated with oral acyclovir, and none of these patients had pregnancy complications. Overall, this cohort did not have a noticeably increased rate of any birth complication compared to the general population.^15,18^ Women in the cohort who did have birth complications trended toward having earlier onset of PR, at 10.75 weeks compared to 15.21 weeks of pregnancy, but did not reach statistical significance (*P* = .3). Of note, limitations of this study were a small sample size and lack of molecular data of HHV 6 or 7 viral loads, which has been previously reported as a risk factor for pregnancy complications associated with PR.^12^

Most recently, a retrospective case-control study compared 31 pregnant patients with PR to 75 pregnant patients without PR matched by age, race, and ethnicity.^20^ Complications observed included SA, chorioamnionitis, meconium passage before delivery, neonatal deformity, and nonreassuring fetal status. Both cohorts exhibited similar rates of birth complications, including a 13% SA rate in both PR patients and controls, aligning closely with pooled risk rates in larger pregnancy studies.^21^ Notably, there was no significant difference in PR onset between patients with and without complications (12.5 weeks vs 14.9 weeks,*P* = .48). Treatment for PR varied among patients, including topical steroids, oral acyclovir, or over-the-counter creams. Two PR patients without complications received oral acyclovir, while no PR patients with complications received this medication. Antiviral treatment may have impacted their outcomes, possibly decreasing the likelihood of complications.^22,23^

Multiple studies have suggested the clinical relevance of HHV 6 titers in the outcomes of PR-associated pregnancies. HHV 6 has been shown to be inherited from parent to child via chromosomally integrated HHV 6 (iciHHV 6) caused by the reactivation of HHV 6 within the pregnant individual.^11,24,25^ One study also noted that mothers with iciHHV 6 were more likely to undergo an SA, with SA rates in HHV 6-positive mothers at 27.6% compared to 14.5% in HHV 6-negative mothers.^25^ Another study demonstrated embryonic tissue findings from a 19-week intrauterine fetal death compatible with HHV 6-induced damage, with the level of HHV 6 DNA load in the embryonic tissue (60 copies/10^6^ cells) comparable with the one found in the mother’s skin lesions (80 copies/10^6^ cells).^10^ Therefore, it is possible PR may represent a systemic HHV 6 infection with the risk of transmission of the virus from mother to fetus, implicating a potential mechanism of SA in pregnant individuals with higher viral loads of HHV 6. As suggested by Monastirli et al.,^26^ the clinician may consider the assessment of HHV 6 DNA via nested PCR in pregnant patients with suspected PR with positive findings potentially warranting antiviral therapy, though PCR result turnaround times and costs may limit the practical use of this test.

To better understand the current knowledge of treatments and interventions for PR in the general population, Contreras-Ruiz et al.^23^ compiled 14 relevant trials for a total of 761 participants. Overall, treatment with acyclovir was associated with improvement in the exanthem compared to placebo or no treatment. When compared to a clinical trial of acyclovir (400 mg 5 times daily for 7 days) in 64 patients with PR, a case series of 3 patients treated with valacyclovir (1 g 3 times daily for 7 days) was noted to improve PR as significantly as acyclovir treatment.^27^ In a separate review, Mahajan et al.^22^ compared the use of erythromycin and acyclovir for PR in 2 studies comprising over 70 patients; acyclovir provided greater pruritis relief and faster resolution of lesions compared to erythromycin.^22^ The optimal dosage of acyclovir and valacyclovir for PR treatment remains unknown; however, a clinical trial of 64 patients with PR demonstrated that low-dose acyclovir (400 mg 5 times daily for 7 days) was as effective in decreasing exanthem duration and pruritus as when compared to a study of 87 patients treated with high-dose acyclovir (800 mg 5 times daily for 7 days).^27–29^

Mahajan et. al.^22^ suggest limiting systemic therapies for PR in pregnant patients,^13^ though for severe cases, oral acyclovir has been reported as efficacious.^30^ Although controlled studies evaluating the safety of acyclovir and valacyclovir in pregnancy are lacking, multiple studies, including over 1,800 patients have found that acyclovir or valacyclovir exposure in the first trimester of pregnancy—when PR may be of highest risk—was not associated with an increased risk of major birth defects.^31–33^ Clinical recommendations for providers are summarized in Table 3.

**Table 3:** Clinical recommendations for providers

Clinical recommendations for providers:	
Recommendation	Rationale
Pregnant women with suspected PR should have syphilis ruled out. Drug reactions and other differential diagnoses should also be explored.	It is important to consider other potential causes of rash, especially those with potential for fetal harm, to ensure accurate diagnosis and appropriate treatment.
For pregnant patients presenting with PR with onset before 15 weeks, involvement of extensive body surface area, constitutional symptoms, or high HHV 6 viral load, antiviral therapy should be considered.	These factors may indicate a higher risk for spontaneous abortion.
Consider utilizing acyclovir (400 mg 5 times daily for 7 days) or valacyclovir (1 g 3 times daily for 7 days) as a treatment for PR with high-risk features in pregnancy.	Acyclovir and valacyclovir have shown the highest effectiveness in treating PR and are generally considered safe in pregnancy, including first trimester.
For pregnant patients with PR onset after 15 weeks and a milder disease course, outcomes are reassuring and patients should be counseled accordingly.	The prognosis for PR in later pregnancy and milder cases is generally better.

HHV, human herpesvirus; PR, pityriasis rosea.

Our study is subject to several limitations. There is minimal literature available on PR in pregnancy, and inconsistent data reporting across the reviewed studies. Multiple studies included only 1 to 2 patients.^2,14–17^ In those with larger cohorts, the data recorded and reported was often inconsistent. Two studies reported only individual data for patients with unfavorable outcomes or perinatal problems.^12,18^ Other studies omitted data on PR onset and HHV 6 viral loads.^2,18–20^ Additionally, studies varied on their characterization of an “unfavorable” pregnancy outcome. To control for these issues, we extrapolated overall outcome totals and analyzed individual data where available, and additionally created our own definition of an “unfavorable outcome” as defined in our methodology. The retrospective nature of the included studies and the nonspecific, often transient nature of PR—which may lead to underdiagnosis—pose further challenges in accurately assessing the condition’s impact on pregnancy outcomes. The effect of treating PR in pregnancy is difficult to investigate due to the ethical issues in performing clinical trials in pregnant women. In the studies reviewed above, several pregnant patients took antivirals with no reported negative treatment side effects.^31–33^ It is unknown if treatment of their PR impacted birth outcomes. While we have attempted to control for these factors, such variability in data quality may affect conclusions, underscoring the need for more expansive research to elucidate the relationship between PR in pregnancy and maternal-fetal outcomes.

## Conclusion

Conflicting data exists regarding the impact of PR on pregnancy outcomes, with varying reports on its influence and cases of healthy births. Notably, when PR emerges within the first 15 weeks of pregnancy, covers a larger body surface area, is linked to constitutional symptoms, or presents with a higher HHV 6 viral load, the risk of adverse outcomes, especially SA, may increase, making these conditions potential prognostic factors for fetal health and an important area of further research to clarify treatment guidelines. Conversely, PR onset later in pregnancy is not strongly associated with adverse outcomes. Limited long-term outcomes data for children born to PR-affected mothers suggests that initially observed unfavorable outcomes may be temporary. Given the transient nature of most reported adverse effects, we advocate that the main concern for this patient population would be increased rates of SA.

Though a direct causal association cannot be elucidated, and despite potential biases in the available data, there is a potential detriment of PR in pregnancy. As such, we recommend close follow-up and consideration of antiviral treatment for pregnant PR patients with high-risk factors. As acyclovir and valacyclovir may treat PR and are generally considered safe in pregnancy, including during the first trimester, we recommend these agents when treating. It is also recommended that syphilis is ruled out in all pregnant women with suspected PR, regardless of severity. In all cases, patient counseling, including the risks and benefits of treatment, and shared decision-making is vital.

## Conflicts of interest

The authors made the following disclosures: M.K.P. receives royalties from UpToDate, is a member of the scientific advisory board of Proctor & Gamble, and has served as a consultant for Incyte. S.R.L. has served as a consultant for Moberg Pharmaceuticals, BelleTorus Corporation, Eli Lilly, and Ortho-Dermatologics. The other authors have no conflicts of interest.

## Funding

None.

## Study approval

N/A

## Author contributions

SM, CSO, MKP and AKB: Performed conceptualization, literature search, data analysis, and original draft writing. All authors helped to review, revise, and edit the manuscript. All authors have confirmed the originality of this paper and have approved of the final version of this manuscript.
